# Optically Transparent Ferromagnetic Nanogranular Films with Tunable Transmittance

**DOI:** 10.1038/srep34227

**Published:** 2016-09-28

**Authors:** Nobukiyo Kobayashi, Hiroshi Masumoto, Saburo Takahashi, Sadamichi Maekawa

**Affiliations:** 1 Research Institute for Electromagnetic Materials, 2-1-1, Yagiyama-minami, Taikaku-ku, Sendai 982-0807, Japan; 2 Frontier Research Institute for Interdisciplinary Sciences, Tohoku University, 6-3, Aramaki aza Aoba, Aoba-ku, Sendai 980-8578, Japan; 3 Institute for Materials Research, Tohoku University, 2-1-1 Katahira, Aoba-ku, Sendai 980-8577, Japan; 4 Advanced Science Research Center, Japan Atomic Energy Agency, 2-4, Shirakata Shirane, Tokai-mura, Naka-gun, Ibaraki-ken 319-1195, Japan

## Abstract

Developing optically transparent magnets at room temperature is an important challenge. They would bring many innovations to various industries, not only for electronic and magnetic devices but also for optical applications. Here we introduce FeCo-(Al-fluoride) nanogranular films exhibiting ferromagnetic properties with high optical transparency in the visible light region. These films have a nanocomposite structure, in which nanometer-sized FeCo ferromagnetic granules are dispersed in an Al-fluoride crystallized matrix. The optical transmittance of these films is controlled by changing the magnetization. This is a new type of magneto-optical effect and is explained by spin-dependent charge oscillation between ferromagnetic granules due to quantum-mechanical tunneling.

Magnets with transparency to light are very promising for new applications. Various transparent magnetic materials have been proposed (e.g., magnetic semiconductors doped with ferromagnetic elements such as Co^1^,^2^,^3^ and nanocrystalline iron oxides such as magnetite (Fe_3_O_4_)^4^ and hematite (Fe_2_O_3_)^5^,^6^). However, materials with large magnetization and high optical transparency at room temperature have not yet been realized. In semiconductors, either the magnetization is too small at room temperature to be useful for applications or the magnetic transition temperatures are too low. If iron oxides have strong magnetization, the optical transparency is low (Fe_3_O_4_); and if they have high optical transparency, the magnetization is very weak (Fe_2_O_3_). On the other hand, magnetorefractive effect in nanogranular films and multilayers with giant magnetoresistance (GMR) has been reported^7^,^8^,^9^,^10^,^11^. Magnetorefractive effect is a magneto-optical effect due to GMR.

In this study, we present FeCo-(Al-fluoride) nanogranular films exhibiting ferromagnetic properties with high optical transparency in the visible light region. Optical transmittance is controlled by changing the magnetization. This is a new magneto-optical effect that is explained by the tunneling magneto-dielectric (TMD) effect^12^,^13^.

Nanogranular films consisting of nanometer-sized magnetic metal granules and a ceramic insulating matrix exhibit various functional properties depending on the composition ratio of the two elements, granules to matrix^14^,^15^. Because dielectric and optical properties are intimately correlated^16^, there is significant interest in the optical properties of nanogranular films with the TMD effect. In addition, these films have significant practical advantages (e.g., they are easily fabricated and are thermally stable^17^,^18^, and have been applied in magnetic sensors^19^,^20^).

## Results

### Optical and magnetic properties of FeCo-(Al-fluoride) nanogranular films

**Figure 1:**
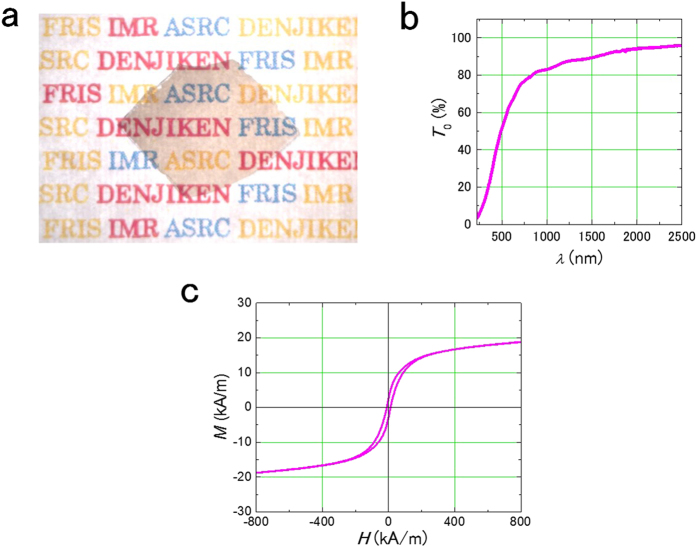
Optical transmittance and magnetic properties of Fe_9_Co_5_Al_19_F_67_ film. (**a**) Photograph of Fe_9_Co_5_Al_19_F_67_ film deposited on the 660 °C glass substrate (Corning Eagle 2000). Film thickness is 1 μm. Film is transparent; and the red, blue, and yellow letters behind the film are seen clearly. (**b**) Transmittance vs. wavelength of Fe_9_Co_5_Al_19_F_67_ film depicted in Fig. 1a. This measurement is in the wavelength range of ultraviolet (400 nm) to near infrared (2000 nm). Using FTIR, only the film transmittance was measured with reference to the substrate glass at the same time. (**c**) The magnetization curve of Fe_9_Co_5_Al_19_F_67_ film presented in Fig. 1a. This magnetization curve has hysteresis with coercivity of 12 kA/m and magnetization of 18 kA/m, indicating that this film is ferromagnetic.

Figure 1a is a photograph of a Fe_9_Co_5_Al_19_F_67_ film deposited on a glass substrate (Corning Eagle 2000) heated to 660 °C. The film is about 1 μm thick. Red, blue and yellow letters behind the thin film are seen clearly. Figure 1b shows the dependence of the transmittance on the light wavelength in the Fe_9_Co_5_Al_19_F_67_ film presented in Fig. 1a. This film has substantial optical transmittance even for short wavelengths less than 400 nm, which is the limit that can be measured in this experiment, and exhibits a high transmittance of 90% to light of wavelength 1500 nm, which is in the band for optical communications. The magnetization curve of the Fe_9_Co_5_Al_19_F_67_ film is presented in Fig. 1c. The film exhibits hysteresis, and the magnetization is 18 kA/m, confirming that the film has both good optical transmittance and ferromagnetic properties.

**Figure 2:**
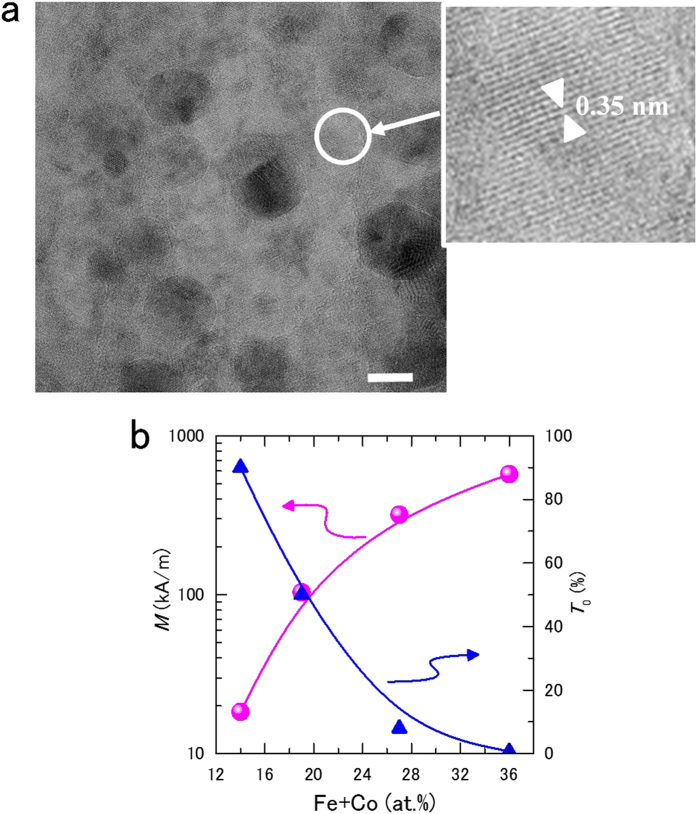
Nanogranular structure and composition dependence of FeCo-(Al-fluoride) film. (**a**) High-resolution transmission electron microscope image obtained from Fe_9_Co_5_Al_19_F_67_ film. The lattice spacing of AlF_3_(012) in 0.35 nm. (scale bar, 10 nm) (**b**) Relationship between the amount of Fe + Co in the films and the magnetization and transmittance of 1 μm thick film. These films were deposited on substrates at 660 °C. As Fe + Co increases, magnetization increases and transmittance decreases.

Figure 2a shows a high-resolution transmission electron microscope image obtained from the Fe_9_Co_5_Al_19_F_67_ film depicted in Fig. 1. This film consists of FeCo magnetic alloy of nanometer-sized granules dispersed in an Al-fluoride matrix. This micrograph has many dark circles with diameters ranging from 10 to 15 nm. In addition, a bright section covers the whole area. The dark circles are FeCo alloy granules, and the bright section with a lattice pattern indicates the Al-fluoride matrix with AlF_3_ crystal structure.

Fluoride crystals (e.q., MgF_2_ and BaF_2_) have good transmittance and are widely used as optical materials. AlF_3_ crystals also exhibit good transmittance from the short-wavelength region (200 nm) to near-infrared (2000 nm). On the other hand, FeCo is a ferromagnetic alloy with the largest known magnetization^21^. FeCo alloy granules with diameters exceeding 10 nm exhibit ferromagnetism because the granules are larger than the superparamagnetic critical diameter^22^ at room temperature. However, since the diameter of the granules is very small compared to the light wavelength, light can pass through the film (to be discussed later). If the density of the FeCo granules in the film increases, transmittance decreases (Fig. 2b). This behavior can be explained simply since the FeCo granules are of the origin of the ferromagnetic properties while the Al-fluoride matrix allows optical transparency.

**Figure 3:**
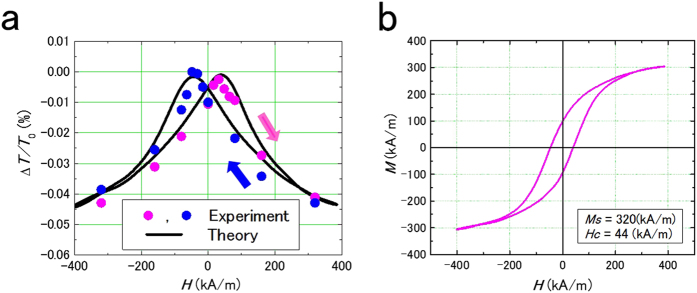
Magneto-optical effect of Fe_13_Co_10_Al_22_F_55_ film. (**a**) Change in the transmittance of the light wave length of 1500 nm of Fe_13_Co_10_Al_22_F_55_ film deposited at 600 °C. The dots denote the experiment results of Δ*T/T*_0_. The solid lines denote the theoretical results as a function of magnetic field *H*, where the magnetization curve in Fig. 3b is used for *m* = *M*(*H*)/*M*_400_ and *M*_400_ is the magnetization at the maximum measured magnetic field of 400 kA/m. (**b**) Magnetization curve of Fe_13_Co_10_Al_22_F_55_ film. Transmittance decreases with increased magnetic field. This result indicates that transmittance is controlled by magnetization.

Figure 3a depicts the change in the transmittance (Δ*T/T*_0_) of light with wavelength of a 1500 nm, Fig. 3b presents the magnetization curve of the Fe_13_Co_10_Al_22_F_55_ film. Transmittance decreases with an increase of magnetic field. The hysteresis of the transmittance is caused by the magnetization, as seen in Fig. 3. Here, Δ*T* = *T*_*M*_−*T*_0_, where *T*_*M*_ is the transmittance with the magnetization *M*, and *T*_0_ is that with zero magnetization. Table 1 lists *ΔT/T*_0_, the magnetization and the transmittance in Fe_9_Co_5_Al_19_F_67_ (Fe + Co = 14 at.%) and Fe_13_Co_10_Al_22_F_55_ (Fe + Co = 23 at.%) films. Δ*T/T*_0_ is observed in both films. It is noteworthy that optical transmittance changes with the magnetic field (Δ*T/T*_0_  = 0.03% and 0.05%). As indicated in Fig. 3a, the magnetic fields at which two of the maxima in the transmittance appear are consistent with the coercivity. This result clearly confirms that the change in the transmittance corresponds to magnetization. The DC resistivity of the films shown in Fig. 3 and Table 1 is larger than 10^11^ μΩ m and the magnetoresistance was not observed. The result in Fig. 3 and Table 1 demonstrate a new magneto-optical effect in transparent nanogranular films.

### Mechanism of optical transmission responses in nanogranular films

**Figure 4:**
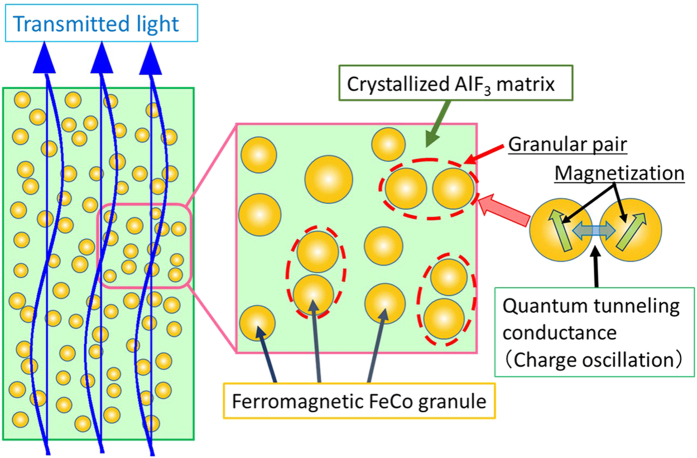
Image of optical transmission and magneto-optical response of a nanogranular film. Schematic of a nanogranular film with nanometer-sized granules dispersed in an insulator matrix. The right side of Fig. 4 depicts a schematic profile of two granules. In an AC electric field, the oscillating transition of an electric charge carrier occurs due to the quantum-mechanical tunneling. Tunneling depends on the relative orientation of the magnetization of the granules. This spin-dependent charge oscillation induces the magneto-optical response of the nanogranular films.

Optical transmission responses to magnetization in nanogranular films may be explained by the TMD effect. Figure 4 illustrates a nanogranular structure with the image of optical transmittance and a model of a granular pair. The magneto-optical response is due to transition of electric charges between neighboring ferromagnetic granules through an insulating barrier via quantum-mechanical electron tunneling^23^,^24^, which depends strongly on the relative orientation of magnetization of the granules. When optical light is incident on the film, electric charge carriers in granules are subject to the oscillating electric field of the light that causes tunneling of the charge carriers back and forth between neighboring granules through the thin insulator barrier (Fig. 4). The oscillation of charging states between granules is spin-dependent and contributes to additional magneto-dielectric and optical responses of nanogranular films^24^.

Incorporating the TMD constant^12^ with a broad distribution of dielectric relaxation around the characteristic relaxation time^24^,^25^ 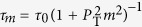, where *P*_T_ is the tunneling spin polarization, *m* = (*M*/*M*_*s*_) is the normalized magnetization and *M*_*s*_ is the saturation magnetization, we have the total magneto-dielectric constant of granular films


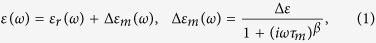


where *ε*_*r*_(*ω*) is the effective dielectric constant of the media in the absence of tunneling effect between granules, Δ*ε*_*m*_(*ω*) is the tunneling contribution^12^, Δ*ε* is the dielectric strength, and *β* is the Cole-Cole’s exponent (0 < *β* ≤ 1) representing a measure of the distribution of relaxation time^26^. In magnetic nanogranular films, *β* = 0.7 to 0.8 was found in a previous study^12^.

Using the dielectric constant (1) in the formula of transmission for a normal-incident optical light through a film^27^, we obtain the magneto-optical transmittance of a granular film as 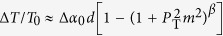, where Δ*α*_0_ is the magneto-optical absorption coefficient and *d* is the film thickness (see Methods for details). In Fig. 3a, we fit the magnetic field dependence of Δ*T/T*_0_ using the experiment data of the magnetization curve in Fig. 3b for the optical light of wave-length *λ* = 1500 nm and frequency *ω* = 10^8^ s^−1^, refractive index *n*_*r*_ = 3, and film thickness *d* = 1000 nm. Using the values of *P*_T_ = 0.5, *β* = 0.7 (*P*_T_ and *β* values are a little different from the previous results^12^. This is because of the increase of the granule size and the granule size distribution as seen in Fig. 2), Δ*ε* = 300 and *τ*_0_ = 10^−8^ s, appropriate for the Fe+Co of 23 at.% granular film^12^ and Δ*α*_0_*d* = (2*πd*/*n*_*r*_*λ*)(*ωτ*_0_)^−β^Δ*ε*sin(β*π*/2) = 2.3×10^−3^ we find a good agreement between the experiment and theoretical data (Fig. 3a), in particular for the hysteretic behavior of the transmittance reflecting the magnetization process in Fig. 3b. The magnetic fields, at which the transmittance is greatest, coincide with the coercive fields where there is a change of sign in the magnetization curves.

The values of Δ*T/T*_0_ can be enhanced if one uses a half-metal with full spin polarization (*P*_F_ = 1) for ferromagnetic nanogranules; makes the granule density higher, which shortens relaxation time due to the reduced distance between granules; and designs broader size distribution, which makes *β* smaller. Nanogranular structures can be controlled by changing the film composition, the deposition conditions, and the annealing. For instance, when the values of *P*_F_ = 1 and *τ*_0_ = 10^−9^ s are used, large magneto-optical transmittances of Δ*T/T*_0_ (~5% for *β* = 0.6 and ~10% for *β* = 0.5 are expected in half-metallic nanogranular films.

## Discussion

We have reported that nanogranular FeCo-(Al-fluoride) films are optically transparent ferromagnetic materials. These films have transmittance even for short wavelengths of light (less than 400 nm), exhibit 90% transmittance at a wavelength of 1500 nm, and are ferromagnetic with magnetization exceeding 18 kA/m at room temperature. Furthermore, these films have magneto-transmittance response Δ*T/T*_0_ of 0.05% at a wavelength of 1500 nm. This new magneto-optical phenomenon is explained by the TMD effect due to the spin-dependent quantum effect in the nanogranular structure. A large value of Δ*T/T*_0_ (more than 10%) is expected theoretically in nanogranular films by optimizing material and structural conditions.

Magnetic materials in electric devices are not optically transparent. With the realization of a transparent magnet, more complete display devices will be constructed. For example, speed and fuel meters and a map can be displayed directly on the front glass of a car or an airplane.

## Methods

### Preparation of thin film samples

Thin films were prepared by a tandem deposition method^28^ using a conventional RF-sputtering apparatus. Sputter deposition was performed on a 50 × 50 mm glass (Corning Eagle 2000) substrate at 600 to 700 °C in argon atmosphere with 1.3 Pa pressure during deposition, using a 76 mm-diameter Fe_60_Co_40_ alloy disk target and an AlF_3_ powder target compacted in the form of a 76 mm-diameter disk.

### Composition and structural analysis

The composition ratio of Fe-Co (granule) and Al-F (matrix) was controlled by changing the RF power applied to each target. The chemical composition of Fe, Co, Al, and F in the thin films was analyzed using wavelength dispersion spectroscopy (WDS). For structural analysis, transmission electron microscopy (TEM) was performed on several selected thin films.

### Measurements of optical and magnetic properties

Optical transmittance was measured using Fourier transform infrared spectroscopy (FTIR) with a measurement waveband of 400 to 2000 nm. Change in the transmittance was measured using an optical spectrometer with a measurement waveband of 900 to 2000 nm and a magnetic field of 0 to 480 kA/m. The magnetization curves were measured using a vibrating sample magnetometer (VSM). In the magnetization and magneto-optical measurements, a magnetic field was applied parallel to the films surface. All the measurements reported in this paper were carried out at room temperature.

### Derivation of the transmittance

The transmittance of the electromagnetic wave incident normal to the plane of a film with the effective dielectric constant *ε (ω*) and thickness *d* is obtained by calculating the Poynting vectors of the incident, reflected, and transmitted electromagnetic waves. The resulting transmittance *T*_*M*_ is expressed as^27^


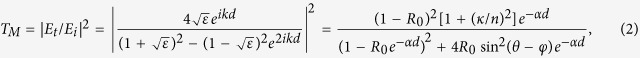


where *E*_*i*_ is the incident electric field, *E*_*t*_ is the transmitted electric field, 
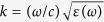
 is the complex wave number, 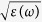 = *n* + *iκ* is the complex refractive index, *α* = 4*πκd*/*λ* is the absorption coefficient, *θ* = 2*πnd*/*λ*, *φ* = −tan^−1^[2*κ*/(*n*^2^ + *κ*^2^−1)], *λ* is the wave length, and *R*_0_ = [(*n*−1)^2^ + *κ*^2^]/[(*n* + 1)^2^ + *κ*^2^].

The interference is weak (Fig. 1b), due to modulation of film thickness and/or refractive index, which allows us to average *T*_*M*_ over (*θ*−*φ*) from 0 to 2*π*, yielding^25^


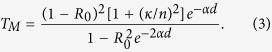


The effective dielectric constant of granular films may be separated into the two contributions


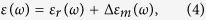


where *ε*_*r*_(*ω*) is the effective dielectric constant in the absence of the tunneling effect between granules and Δ*ε*_*m*_(*ω*) is the tunneling contribution of the form^12^,


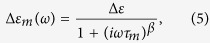


where Δ*ε* is dielectric strength; *τ*_*m*_ is the characteristic relaxation time given by the spin-dependent tunneling rate^24^,^25^ 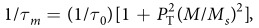 where *P*_T_ is the tunneling spin polarization, *M* is the magnetization and *M*_*s*_ is the saturation magnetization; and *β* is an exponent representing a measure of the distribution of relaxation time^26^ (*β *~ 0.7 to 0.8 in the granular films^12^). In the optical region, the light frequency (~10^15^ s^−1^) is much higher than the tunneling rate (~10^4^ s^−1^ to 10^9^ s^−1^) depending on the ferromagnetic composition^12^ (ωτ_*m*_≫1) so that the tunneling contribution is approximated as Δ*ε*_*m*_(*ω*) ≈ Δ*εe*^−*iβπ*/2^(*ωτ*_*m*_)^−*β*^.

In the optical region (ωτ_*m*_≫1), the refractive index 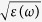 can be expanded with respect to the tunneling contribution Δ*ε*_*m*_(*ω*) as


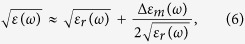


where


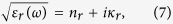



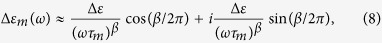


The real and imaginary parts of the complex refractive index are written as


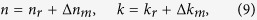


where


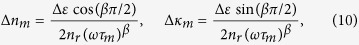


in the highly transparent region (*k*_r_/*n*_*r*_)^2^≪1.

It follows from Eqs (3), (9) and (10) that the dominant contribution to the magneto-optical effect arises from the magneto-optical part Δ*α*_*m*_ of the absorption coefficient *α* = *α*_*r*_ + Δ*α*_*m*_, where *α*_*r*_ = 4*πκ*_*r*_/*λ* and Δ*α*_*m*_ = 4*π*Δ*κ*_*m*_/*λ*, yielding the transmittance


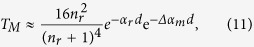


as a function of applied magnetic field *H* through the magnetization curve *M*(*H*). Therefore, the magneto-transmittance effect of a granular film is obtained as


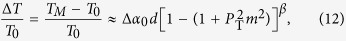


where Δ*α*_0_ = (2*π*/*n*_*r*_*λ*)(*ωτ*)^−β^Δ*ε*sin(β*π*/2) and *m* = *M*(*H*)/*M*_*s*_. Equation (12) is used to analyze the experimental results of the magneto-optical transmittance ratio Δ*T/T*_0_ versus applied magnetic field *H* in the Fe_13_Co_10_Al_22_F_55_ (Fe+Co = 23 at.%) film, as illustrated in Fig. 4.

## Additional Information

**How to cite this article**: Kobayashi, N. *et al.* Optically Transparent Ferromagnetic Nanogranular Films with Tunable Transmittance. *Sci. Rep.* **6**, 34227; doi: 10.1038/srep34227 (2016).

## Figures and Tables

**Table 1:** List of optical and magnetic properties.

Sample (Substrate temp.)	Δ*T/T*_0_	Magnetization	Transmittance (1500 nm)
Fe_9_Co_5_Al_19_F_67_ (660 °C)	0.03%	18 kA/m	90%
Fe_13_Co_10_Al_22_F_55_ (600 °C)	0.05%	320 kA/m	20%

Δ*T/T*_0_, magnetization and transmittance of Fe_9_Co_5_Al_19_F_67_ and Fe_13_Co_10_Al_22_F_55_ film. Transmittance and Δ*T/T*_0_ is the value of light wave length in 1500 nm.
